# Prognostic Association of the Two-Cycle On-Treatment CRAFITY Score in Advanced HCC: A Single-Center Retrospective Cohort Study

**DOI:** 10.3390/curroncol33100598

**Published:** 2026-10-02

**Authors:** Xiaochun Chen, Xinkun Guo, Guobin Chen, Susu Zheng, Yanfang Wu, Jingfang Wu, Hong Chen, Zhenzhen Zhang, Boheng Zhang

**Affiliations:** 1 Department of Hepatobiliary Oncology, Zhongshan Hospital (Xiamen), Fudan University, Xiamen 361015, China; 2 Xiamen Clinical Research Center for Cancer Therapy, Xiamen 361015, China; 3 Fujian Provincial Clinical Research Center for Precision Medicine of Abdominal Tumors, Xiamen 361015, China

**Keywords:** hepatocellular carcinoma, immunotherapy, CRAFITY score, AFP, CRP, prognostic biomarker, on-treatment biomarker assessment, real-world study

## Abstract

The baseline CRAFITY score, derived from AFP and C-reactive protein, is a validated prognostic biomarker for hepatocellular carcinoma (HCC) patients receiving immunotherapy. However, whether its prognostic value can be improved by on-treatment reassessment remains unclear. We evaluated the two-cycle on-treatment CRAFITY score in 108 advanced-HCC patients who received immune-based combination therapy as first- or second-line treatment. The score was calculated at baseline and after two cycles. Kaplan–Meier, Cox regression, time-dependent ROC, landmark, and sensitivity analyses were performed. The two-cycle score significantly stratified overall survival (median OS from landmark: 34.6, 12.9, and 6.7 months for scores 0, 1, and 2; *p* < 0.0001) and remained independently associated with OS after adjustment for vascular invasion, treatment line, and concomitant transarterial chemoembolization (TACE) (HR 2.36, 95% CI 1.70–3.29, *p* < 0.001). The baseline score showed only borderline associations (OS *p* = 0.07; PFS *p* = 0.09). The two-cycle score had an apparent AUC of 0.709 (95% CI 0.586–0.832) at 6 months and 0.745 (95% CI 0.658–0.832) at 12 months. The corresponding values for the baseline score were 0.645 and 0.638. Adding the two-cycle score to a clinical reference model increased the 12-month AUC from 0.699 to 0.811 (ΔAUC = 0.112). Bootstrap internal validation yielded an optimism-corrected C-index of 0.698 (95% CI 0.650–0.776). In conclusion, the two-cycle CRAFITY score provides complementary posttreatment prognostic information for early risk stratification, but prospective external validation is required before clinical application.

## Simple Summary

Liver cancer remains one of the deadliest malignancies worldwide, and immune-based combination therapy is now standard for advanced disease. However, responses vary widely, and simple tools for early risk stratification are still lacking. The CRAFITY score, based on two routine blood tests (AFP and C-reactive protein), is widely used to estimate prognosis before treatment, but its value when measured during early treatment is less clear. In this cohort of 108 Chinese patients with advanced hepatocellular carcinoma, the CRAFITY score measured after two treatment cycles provided useful prognostic information, particularly for identifying high-risk patients within the first six months. This low-cost, readily available tool may support early risk stratification, but prospective studies are needed before it can guide treatment decisions.

## 1. Introduction

Hepatocellular carcinoma (HCC) remains one of the most lethal malignancies worldwide, with a rising incidence particularly in regions endemic for hepatitis B virus (HBV) infection [1]. Despite advances in surveillance and early detection, many patients are diagnosed at intermediate-to-advanced stages, where curative options such as surgical resection, liver transplantation, and ablation are no longer applicable [2]. For decades, systemic therapy for advanced HCC was limited to multikinase inhibitors such as sorafenib [3]. The therapeutic landscape has since been transformed by immune checkpoint inhibitors (ICIs). The landmark IMbrave150 trial established atezolizumab plus bevacizumab as the preferred first-line treatment, showing significant improvements in overall survival (OS) and objective response rate compared with sorafenib [4]. Multiple PD-1/PD-L1 inhibitor-based regimens have since been approved, offering new hope for patients with advanced HCC [5,6].

Despite these advances, responses to ICIs remain highly heterogeneous. Up to one-third of patients exhibit primary resistance, and durable responses occur in only a subset [7]. This variability underscores the need for reliable, accessible biomarkers for early risk stratification and identification of patients who may derive limited benefit from immunotherapy [8].

To address this need, Scheiner et al. developed the CRAFITY score (C-reactive protein and alpha-fetoprotein in immunotherapy), a composite index integrating baseline serum CRP (≥1 mg/dL) and AFP (≥100 ng/mL) [9]. In their derivation and external validation cohorts, the CRAFITY score significantly stratified OS, with median survival of 27.6, 11.3, and 6.4 months for scores of 0, 1, and 2, respectively. A subsequent meta-analysis of four studies comprising 786 patients confirmed that higher baseline CRAFITY scores were consistently associated with shorter OS and progression-free survival (PFS), as well as lower response and disease control rates [10]. These findings established the CRAFITY score as a simple, readily available biomarker capturing both systemic inflammation (CRP) and tumor burden (AFP) [11,12].

However, nearly all existing studies have focused on baseline CRAFITY assessment—a single pretreatment measurement that reflects initial disease severity, but cannot capture the dynamic interplay between tumor and immune system under therapeutic pressure [13]. Growing evidence suggests that dynamic changes in serum biomarkers during early treatment may provide complementary prognostic information alongside static baseline values, and recent reviews have emphasized the priority of on-treatment biomarkers in real-world practice [14,15]. A meta-analysis demonstrated that dynamic decreases in AFP following ICI therapy were significantly associated with improved OS and PFS [16]. Similarly, CRP kinetics during treatment have been associated with prognosis in HCC patients receiving immunotherapy [17]. Recognizing the importance of dynamic assessment, recent studies have proposed refined models incorporating early biomarker changes. The CAR grading system (CRAFITY score and AFP response) combined baseline CRAFITY with AFP response (defined as >15% decline within 3 months) and demonstrated improved prognostic discrimination [18]. The CRAFITY-100 RULE integrated baseline CRAFITY with 4-week AFP/PIVKA-II kinetics, yielding clearer survival stratification than the original score alone [19]. Notably, in the CRAFITY-100 RULE cohort, the original CRAFITY score also failed to significantly stratify OS (*p* = 0.267), whereas the dynamic model distinguished three risk groups with distinct prognoses [19], consistent with the present findings.

We recently reported that the on-treatment prognostic nutritional index (PNI) provided additional prognostic information beyond baseline PNI in the same population [20]. However, whether the CRAFITY score—a composite of AFP and CRP reflecting tumor burden and systemic inflammation—exhibits similar prognostic performance remains unexplored. These observations raise a critical clinical question: Does the on-treatment CRAFITY score assessed during early immunotherapy provide additional prognostic information beyond baseline assessment? While complex models combining baseline scores with biomarker kinetics have been explored, the prognostic value of the absolute on-treatment CRAFITY score alone—using only two routine blood tests (AFP and CRP) at a clinically meaningful timepoint—remains largely unexplored, particularly in Chinese HCC populations receiving diverse immunotherapy regimens in real-world practice.

To address this gap, we conducted a retrospective cohort study to evaluate and compare the prognostic value of baseline versus two-cycle on-treatment CRAFITY scores in advanced-HCC patients receiving first-time immunotherapy (including both first-line and second-line settings). We hypothesized that the two-cycle CRAFITY score would provide posttreatment prognostic information and could serve as a tool for early risk stratification, given its ability to provide posttreatment prognostic information. The two-cycle timepoint (approximately 6–8 weeks) was chosen because it coincides with the first scheduled radiological evaluation in routine clinical practice, making our findings directly applicable to real-world clinical decision-making.

## 2. Materials and Methods

### 2.1. Study Design and Population

We performed a retrospective, single-center cohort study at the Department of Hepatobiliary Oncology, Zhongshan Hospital (Xiamen), Fudan University. This study was reported in accordance with the reporting recommendations for tumor marker prognostic studies (REMARK) [21]. Patients with advanced HCC who initiated immune-based combination therapy as first- or second-line systemic treatment at our center between February 2019 and September 2022 were retrospectively screened for eligibility, with the final follow-up completed in November 2025.

The research protocol was reviewed and formally approved by the Institutional Ethics Committee of Zhongshan Hospital, Fudan University (Xiamen) (approval B2019–026), and all procedures were conducted in strict compliance with the Declaration of Helsinki. Because of the retrospective observational design, written informed consent was waived. All patient records were deidentified prior to data extraction.

The cohort was derived from the same institutional database as our previously published work on the prognostic nutritional index [20]. However, the present study addressed a distinct clinical question focused on the CRAFITY score, and applied additional eligibility criteria requiring paired baseline and two-cycle measurements of alpha-fetoprotein and C-reactive protein to enable score calculation. Inclusion and exclusion criteria are summarized below.

Inclusion criteria:

1. Histologically or clinically confirmed unresectable advanced HCC (or recurrent/metastatic HCC) receiving palliative systemic therapy.
2. First-time initiation of an immunotherapy-based combination regimen (PD-1/PD-L1 inhibitor combined with an anti-angiogenic agent), administered as first- or second-line systemic therapy.
3. Prior or concomitant transcatheter arterial chemoembolization (TACE) permitted, without restriction on the number of sessions.
4. Completion of at least 2 cycles of immunotherapy, with available on-treatment AFP and CRP measurements after 2 cycles of treatment (before cycle 3).
5. Complete baseline and follow-up data for survival endpoints (OS and PFS).

Exclusion criteria:

1. Prior exposure to any immune checkpoint inhibitor (anti-PD-1/PD-L1 or anti-CTLA-4).
2. Immunotherapy initiated as third-line or beyond.
3. Adjuvant or prophylactic immunotherapy in the absence of measurable disease.
4. Missing key variables (baseline or two-cycle AFP/CRP, or survival data).
5. Severe comorbidities (e.g., advanced cardiovascular, renal, or respiratory failure) that could independently influence survival.
6. Uncontrolled active infection or severe uncontrolled inflammatory disease at treatment initiation.

**Figure 1:**
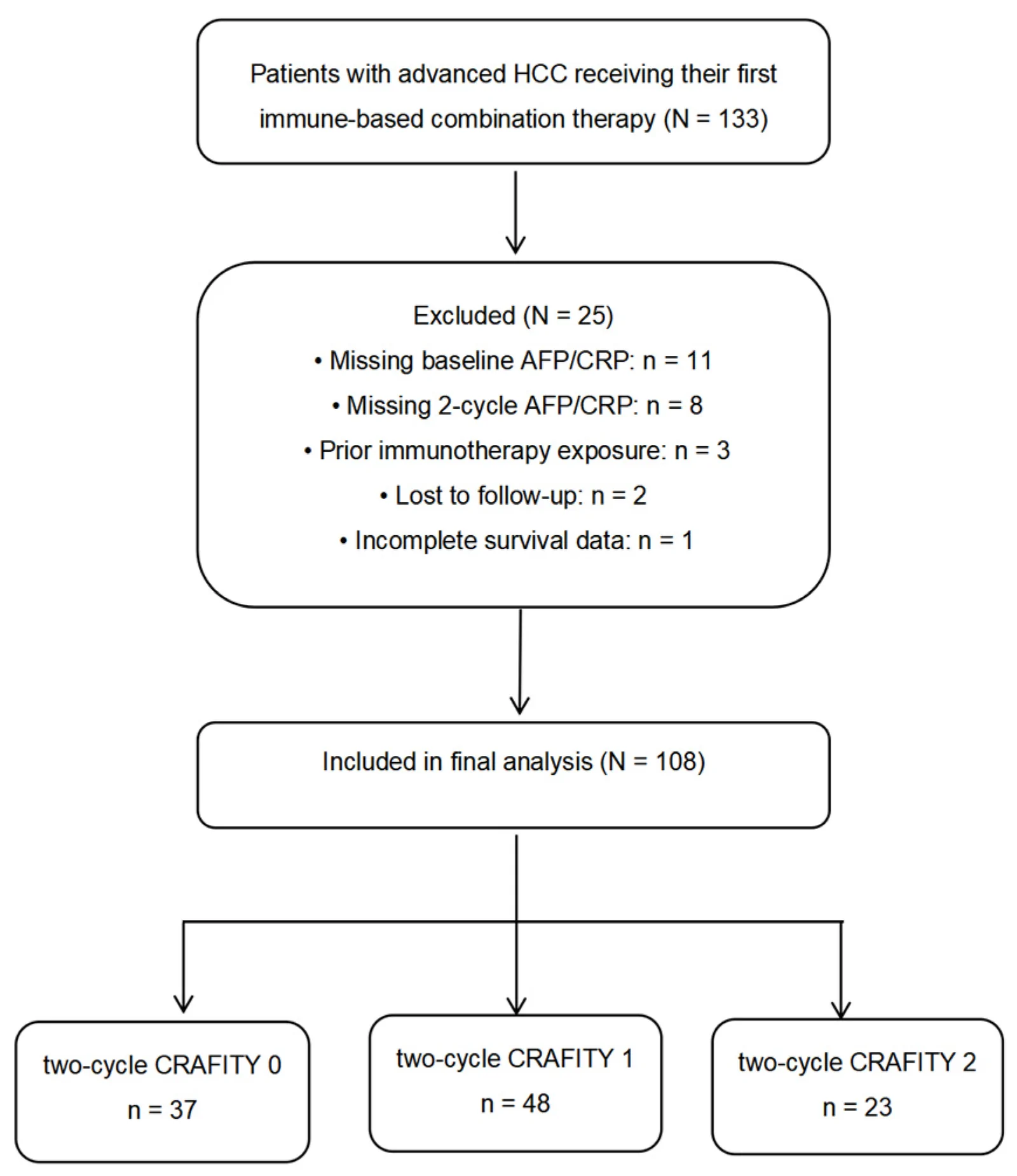
Flowchart of patient selection. A total of 133 patients with advanced hepatocellular carcinoma (HCC) receiving first-time immune-based combination therapy were initially screened. After excluding 25 patients for missing data, prior immunotherapy exposure, loss to follow-up, and incomplete survival data, 108 patients were included in the final analysis and stratified into three groups according to the two-cycle CRAFITY score. Arrows indicate the flow of patient screening and enrollment.

A total of 133 patients with advanced HCC receiving first-time immune-based combination therapy were initially screened. After applying the exclusion criteria, 108 patients were included in the final analysis (Figure 1).

### 2.2. Treatment and Clinical Assessment

All patients received standard-of-care immune-based combination regimens according to National Comprehensive Cancer Network (NCCN) or Chinese Society of Clinical Oncology (CSCO) guidelines [22,23]. The choice of specific PD-1/PD-L1 inhibitors and anti-angiogenic agents was made by the treating physicians based on patient characteristics, tumor burden, liver function, and patient preference.

Serum AFP and CRP levels were measured at baseline (within 7 days prior to the first dose of immunotherapy) and at the two-cycle timepoint, defined as within 1 week before the initiation of the third cycle (i.e., after completion of 2 treatment cycles, approximately 6–8 weeks after treatment initiation). This timepoint coincided with the first scheduled radiological response assessment as per mRECIST, allowing biomarker results and imaging findings to be interpreted concurrently. Patients with uncontrolled active infection or severe uncontrolled inflammatory disease at treatment initiation were excluded. In our practice, if an intercurrent infection develops during treatment, immunotherapy is interrupted until the infection is controlled, and the on-treatment blood sample is collected after resolution. Because concomitant TACE may transiently affect inflammatory biomarkers, a sensitivity analysis excluding all patients who received concomitant TACE during immunotherapy was performed (Section 3.7). Blood samples were collected under standardized fasting conditions and analyzed at the central laboratory of our institution using commercially available assays. The upper limit of detection for AFP was 60,500 ng/mL. Values exceeding this limit were recorded as “>60,500” and assigned a value of 60,501 ng/mL (i.e., the upper limit plus 1) for quantitative analysis. Values below the lower limit of detection (e.g., CRP < 0.3 mg/L) were assigned half the lower limit (0.15 mg/L).

Radiological response was assessed with contrast-enhanced CT or MRI at baseline and at 6- to 8-week intervals during treatment, with response categories defined by modified Response Evaluation Criteria in Solid Tumors (mRECIST) [24].

### 2.3. CRAFITY Score Calculation

The CRAFITY score was calculated according to the original definition proposed by Scheiner et al. [9]:

AFP ≥ 100 ng/mL → 1 point

CRP ≥ 10 mg/L (equivalent to 1 mg/dL, as per the original definition) → 1 point

The total score ranges from 0 to 2 points.

Both baseline CRAFITY score (using pretreatment AFP and CRP) and two-cycle CRAFITY score (using on-treatment AFP and CRP measured after 2 cycles, before cycle 3) were calculated for each patient.

To explore the prognostic value of dynamic changes, ΔCRAFITY was defined as a binary variable indicating whether the two-cycle CRAFITY score decreased compared with baseline (1 = improvement, 0 = non-improvement).

### 2.4. Study Endpoints

The primary endpoint was overall survival (OS), defined as the time from the date of first immunotherapy administration to the date of death from any cause. Patients alive at the last follow-up were censored at the date of last contact.

The secondary endpoints included: progression-free survival (PFS), defined as the time from treatment initiation to the first documented disease progression (per mRECIST) or death from any cause, whichever occurred first; and time-dependent prognostic discrimination of the two-cycle CRAFITY score, assessed using time-dependent receiver operating characteristic (ROC) analysis.

### 2.5. Statistical Analysis

Continuous variables are presented as means ± standard deviation (SD) or medians with interquartile range (IQR), as appropriate. Categorical variables are expressed as frequencies and percentages. Comparisons between CRAFITY score groups (0, 1, and 2) were performed using one-way analysis of variance (ANOVA) or Kruskal–Wallis test for continuous variables and Pearson’s chi-square test or Fisher’s exact test for categorical variables, as appropriate.

Survival curves were generated using the Kaplan–Meier method, and between-group differences were compared by log-rank test. Median survival with 95% confidence interval (CI) was calculated for each subgroup.

Univariable and multivariable Cox proportional hazard regression models were constructed to identify factors independently associated with OS. Variables with clinical significance (age, gender, BCLC stage, Child–Pugh grade, vascular invasion, treatment line, and concomitant TACE) were included in the multivariable model. The proportional hazard assumption was tested using Schoenfeld residuals and was satisfied. Results are reported as hazard ratios (HRs) with 95% CIs. A total of 83 deaths (76.9%) occurred during follow-up, yielding approximately 12 events per variable in the primary multivariable model (7 covariates) [25], exceeding the recommended minimum of 10 events.

In the primary analysis and sensitivity analyses, OS and PFS were calculated from the date of first immunotherapy administration.

Landmark analysis: To address the potential immortal time bias inherent in on-treatment biomarker analyses—where patients must have survived to the two-cycle timepoint to have on-treatment CRAFITY scores measured—we used a landmark framework as the primary analysis for the two-cycle CRAFITY score [26,27]. The landmark time was set at 1.5 months after treatment initiation, approximately corresponding to the completion of 2 treatment cycles (6–8 weeks). All 108 patients were alive at the landmark and were included in the landmark OS analysis. For the landmark PFS analysis, 2 patients who had documented disease progression before the landmark time were excluded, leaving 106 patients. Survival was calculated from this landmark timepoint onward. Kaplan–Meier curves and log-rank tests were performed using these landmark-adjusted survival times. 

Survival starting points: All analyses involving the two-cycle CRAFITY score, including Kaplan–Meier, Cox regression, and time-dependent ROC, used the landmark time (1.5 months after treatment initiation) as time zero. Baseline CRAFITY analyses used treatment initiation as time zero.

Time-dependent ROC analysis was performed as described by Heagerty and Zheng [28] using the timeROC package in R to evaluate and compare the apparent prognostic discrimination of the baseline and two-cycle CRAFITY scores for OS at 6 and 12 months. For descriptive comparison of baseline and two-cycle scores, time-dependent AUCs were estimated among patients alive at the 1.5-month landmark, with survival time measured from the landmark. The area under the curve (AUC) was calculated for each timepoint. AUC values with 95% confidence intervals are reported for each score separately. Formal pairwise comparison of paired time-dependent AUCs could not be reliably performed because bootstrap resampling did not converge in a substantial proportion of resamples. The numerical difference between scores is therefore reported descriptively. To assess incremental prognostic value beyond conventional clinical variables, a clinical reference model (age, gender, BCLC stage, Child–Pugh grade, vascular invasion, treatment line, and concomitant TACE) was compared with the same model plus either the baseline or the two-cycle CRAFITY score, using 500 bootstrap replications.

Calibration and prediction error: Calibration was assessed at 6 and 12 months using calibration plots, calibration slope and intercept, and Brier scores. Predicted survival probabilities were obtained using the pec package. Calibration slope and intercept were estimated using logistic calibration regression on quintile groups of predicted probabilities. The integrated Brier score (IBS) was calculated to quantify overall prediction error, with the Kaplan–Meier model as reference. Calibration slope, intercept, and IBS were calculated as apparent estimates. Bootstrap optimism correction was performed only for the C-index. These findings therefore support internally validated model performance only and do not establish clinical utility or transportability to other settings. Internal validation of the final multivariable Cox model was performed using 500 bootstrap replications, and the apparent and optimism-corrected C-index values were calculated.

Sensitivity analyses: Two prespecified sensitivity analyses were performed. First, to assess the robustness of findings against TACE-related confounding, all patients who received concomitant TACE during immunotherapy were excluded, and the multivariable analysis was repeated in the TACE-free subset (*n* = 38, Supplementary Table S7). This analysis is exploratory given the small sample. Second, a subgroup analysis stratified by TKI type (lenvatinib vs. other TKIs) was performed to assess the stability of the two-cycle CRAFITY score across different anti-angiogenic regimens (Supplementary Table S8).

All statistical tests were two-sided, and a *p* value < 0.05 was considered statistically significant. All analyses were performed using R software version 4.3.1 (R Foundation for Statistical Computing, Vienna, Austria).

### 2.6. Handling of Missing Data

For the primary analysis, complete case analysis was employed. The proportion of missing data was <15% for all key variables.

## 3. Results

### 3.1. Patient Characteristics

A total of 108 patients with advanced HCC who received first-time immune-based combination therapy were included. Median age was 56.0 years (IQR: 47.0–63.0), and 102 patients (94.4%) were male. Most had BCLC stage C disease (96, 88.9%) and Child–Pugh class A (99, 91.7%). Vascular invasion was present in 63 patients (58.3%). Regarding treatment line, 37 patients (34.3%) received first-line therapy, while 71 patients (65.7%) received second-line therapy. Concomitant TACE during immunotherapy was performed in 70 patients (64.8%).

The distribution of immune-based regimens was as follows. In sum, 107 patients (99.1%) received a PD-1 inhibitor combined with a tyrosine kinase inhibitor (TKI). The PD-1 inhibitors used included camrelizumab *(n* = 38), sintilimab (*n* = 35), tislelizumab (*n* = 22), toripalimab (*n* = 7), pembrolizumab (*n* = 3), and nivolumab (*n* = 2). The TKIs administered were lenvatinib (*n* = 88), sorafenib (*n* = 8), apatinib (*n* = 9), and regorafenib (*n* = 2). One patient (0.9%) received atezolizumab plus bevacizumab. A summary is provided in Supplementary Table S1.

Baseline characteristics stratified by the two-cycle CRAFITY score are summarized in Table 1. Patients with a score of 2 had significantly higher baseline AFP levels and a trend toward elevated CRP levels relative to patients with a score of 0 (AFP: 13,382 vs. 619 ng/mL, *p* < 0.001; CRP: 29.2 vs. 14.5 mg/L, *p* = 0.068), reflecting heavier tumor burden and stronger systemic inflammation within this high-risk subgroup. They also had higher rates of vascular invasion and larger tumors (≥5 cm) (*p* = 0.027 and *p* = 0.031, respectively). No significant differences were observed for age, gender distribution, HBV infection, cirrhosis status, ECOG performance status, BCLC stage, Child–Pugh grade, extrahepatic metastasis, multiple tumors, treatment line, or concomitant TACE.

### 3.2. Prognostic Stratification by the Two-Cycle CRAFITY Score

Patients were stratified into three groups according to the two-cycle CRAFITY score: 0 (*n* = 37), 1 (*n* = 48), and 2 (*n* = 23). Kaplan–Meier analysis in the landmark framework demonstrated that the two-cycle CRAFITY score significantly stratified both overall survival (OS) and progression-free survival (PFS).

**Figure 2:**
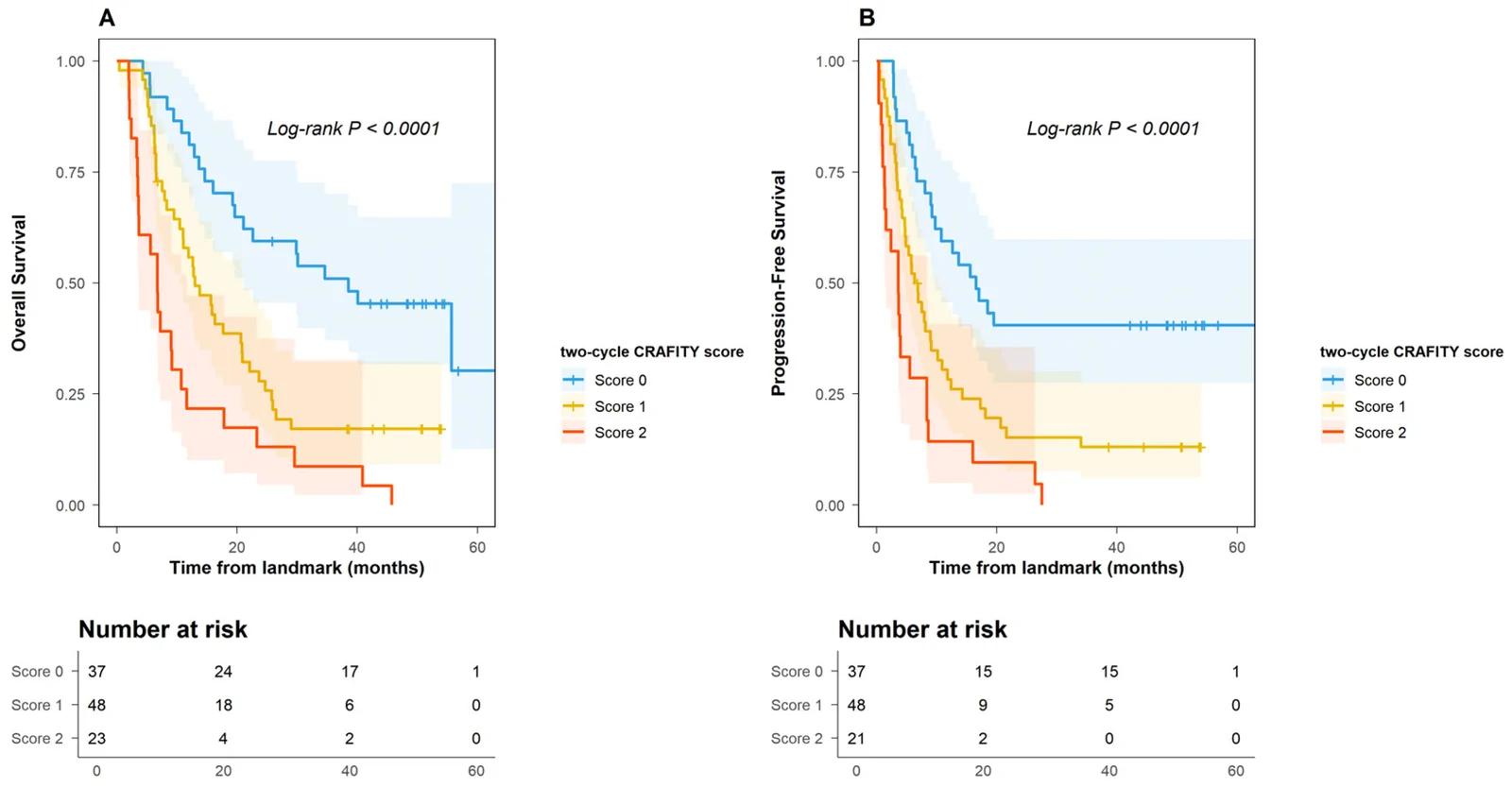
Kaplan–Meier curves for (**A**) overall survival and (**B**) progression-free survival stratified by the two-cycle CRAFITY score, using the landmark time (1.5 months after treatment initiation, approximately corresponding to the completion of 2 treatment cycles) as time zero. All 108 patients were alive at the landmark and included in the OS analysis; 2 patients who progressed before the landmark were excluded from the PFS analysis (*n* = 106). The risk tables below each panel show the number of patients at risk at each timepoint. Shaded areas represent 95% confidence intervals.

The median OS from landmark for patients with scores of 0, 1, and 2 was 34.6, 12.9, and 6.7 months, respectively (log-rank *p* < 0.0001; Figure 2A). Similarly, the median PFS from landmark was 16.6, 6.6, and 3.6 months, respectively (log-rank *p* < 0.0001; Figure 2B).

All two-cycle CRAFITY analyses used the landmark time (1.5 months after treatment initiation) as time zero. All 108 patients were alive at the landmark and were included in the OS analysis (83 events); 2 patients who experienced disease progression before the landmark were excluded from the PFS analysis, leaving 106 patients (84 events).

To explore whether the prognostic value of the two-cycle CRAFITY score differed by treatment line, subgroup analyses were performed. In the first-line subgroup (*n* = 37), the median OS for patients with CRAFITY scores of 0, 1, and 2 was 40.4, 13.4, and 9.1 months, respectively (log-rank *p* < 0.001). In the second-line subgroup (*n* = 71), the corresponding median OS values were 30.1, 11.0, and 6.7 months, respectively (log-rank *p* < 0.001). These findings indicate that the two-cycle CRAFITY score provided significant prognostic stratification in both first-line and second-line treatment settings. Detailed results are presented in Supplementary Table S2. However, the sample size in each subgroup was limited, and these findings should be interpreted as exploratory.

### 3.3. Prognostic Value of the Baseline CRAFITY Score

**Figure 3:**
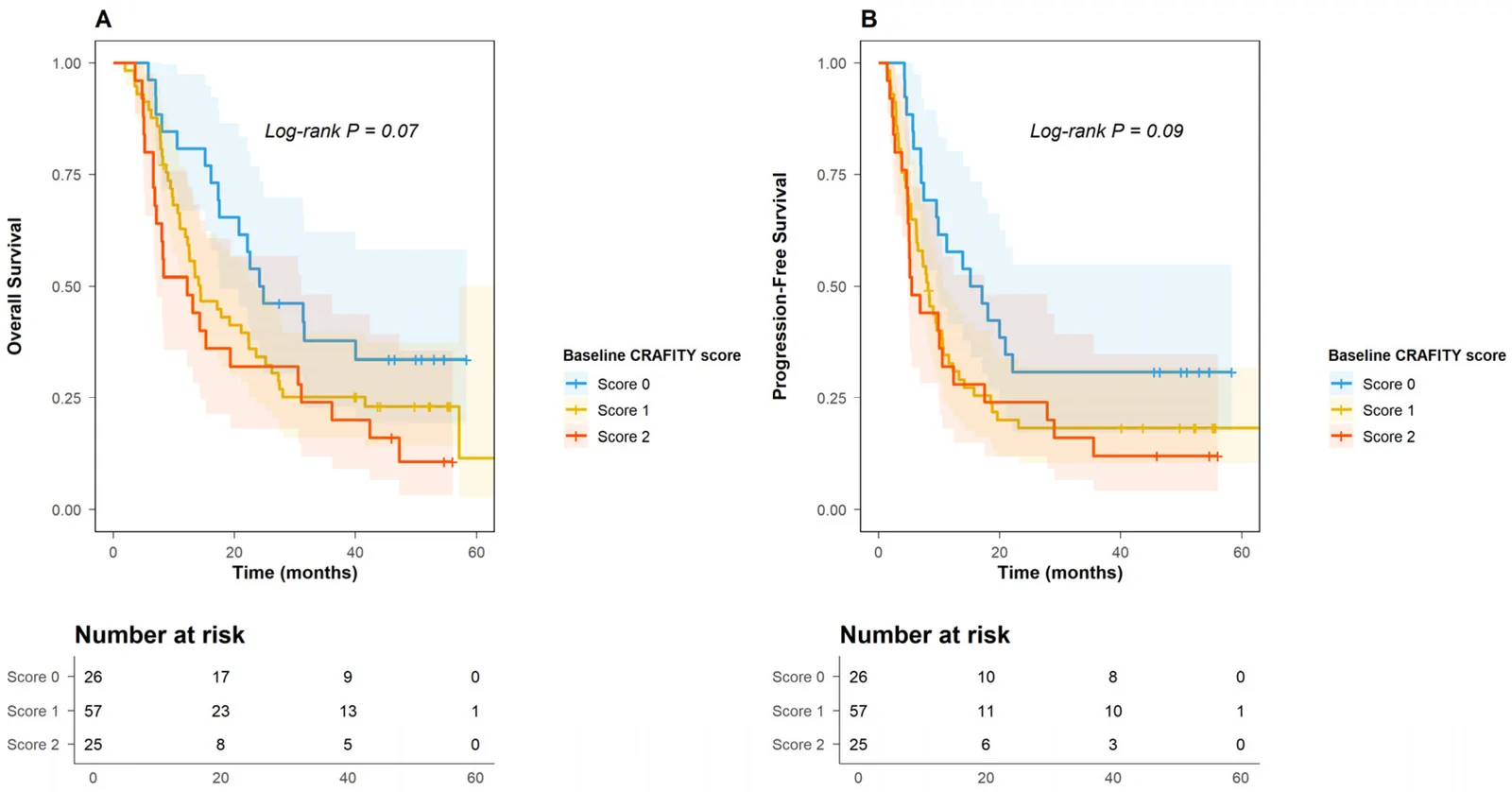
Kaplan–Meier curves stratified by the baseline CRAFITY score. (**A**) Overall survival; (**B**) progression-free survival. The baseline score showed only borderline associations with both OS (log-rank *p* = 0.07) and PFS (log-rank *p* = 0.09), with no statistically significant stratification effect.

Separately, we assessed the baseline CRAFITY score, which did not significantly stratify OS or PFS in this cohort. For OS, the baseline score showed only a borderline association (log-rank *p* = 0.07; Figure 3A). For PFS, the between-group difference did not reach significance either (log-rank *p* = 0.09; Figure 3B). These findings suggest that static pretreatment assessment may provide limited prognostic information in our population. The baseline CRAFITY score has been validated in the original cohort [9], and its prognostic value may vary across different treatment settings and populations. Our results indicate that the two-cycle CRAFITY score may provide posttreatment prognostic information and could serve as a tool for early risk stratification, warranting further prospective investigation.

### 3.4. Association with Early Radiological Response

We further explored the association between the two-cycle CRAFITY score and early radiological response assessed by mRECIST at the two-cycle timepoint (Supplementary Table S3). The objective response rates (ORRs) for scores 0, 1, and 2 were 13.5%, 10.4%, and 8.7%, respectively (*p* for trend = 0.549). The absence of a significant ORR trend may reflect the limited number of objective responses in this cohort. Disease control rates (DCRs) showed a significant decreasing trend: 94.6%, 77.1%, and 69.6%, respectively (*p* for trend = 0.011). These findings are consistent with the survival data, suggesting that lower on-treatment CRAFITY scores were associated with higher disease control rates.

### 3.5. Univariable and Multivariable Cox Regression Analysis

Overall, 42 patients (38.9%) showed improvement in in CRAFITY score at the two-cycle timepoint, while 66 patients (61.1%) showed no improvement (stable or increased score). On univariable analysis, the two-cycle CRAFITY score was significantly associated with OS (HR 2.23, 95% CI 1.66–3.01, *p* < 0.001), whereas the baseline CRAFITY score showed only marginal significance (HR 1.44, 95% CI 1.05–1.96, *p* = 0.02). The discrepancy between the log-rank test (*p* = 0.07) and univariable Cox analysis (*p* = 0.02) for the baseline score likely reflects differences in the statistical hypotheses tested and the limited sample size. The baseline score was therefore not considered a robust prognostic factor in this cohort. The simple directional change of the score (improvement vs. non-improvement) was not significantly associated with OS (HR 0.67, 95% CI 0.39–1.15, *p* = 0.142).

Multivariable Cox regression analysis, adjusting for age, gender, BCLC stage, Child–Pugh grade, vascular invasion, treatment line, and concomitant TACE, demonstrated that the two-cycle CRAFITY score was independently associated with overall survival (HR = 2.36, 95% CI: 1.70–3.29, *p* < 0.001). None of the other covariates reached statistical significance (Table 2). This analysis was performed within the landmark framework among patients at risk at 1.5 months, with follow-up measured from the landmark. The hazard ratio was consistent with the primary analysis, which is expected because all patients included in the OS analysis survived to the landmark and only a common constant (1.5 months) was subtracted from their follow-up times.

Internal validation of the final multivariable model using 500 bootstrap replications yielded an apparent C-index of 0.706 and an optimism-corrected C-index of 0.698 (95% CI 0.650–0.776), indicating minimal optimism.

### 3.6. Time-Dependent ROC Analysis

**Figure 4:**
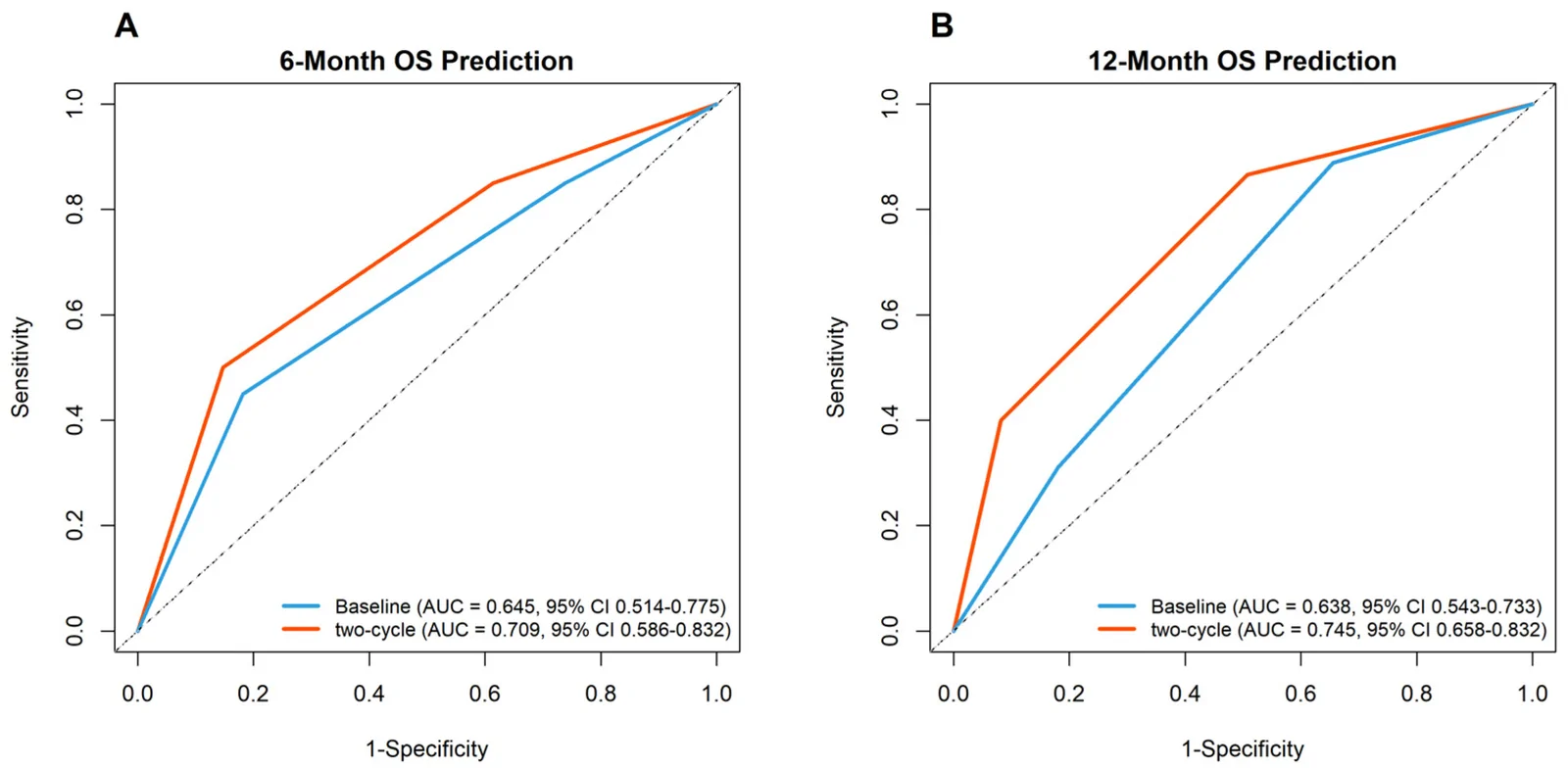
Time-dependent receiver operating characteristic (ROC) curves for (**A**) 6-month and (**B**) 12-month overall survival prediction in the landmark framework. The two-cycle CRAFITY score is shown in red and the baseline score in blue. The dashed diagonal line represents the reference line corresponding to an AUC of 0.5 (no predictive discrimination). AUC values with 95% confidence intervals are shown in each panel. Formal pairwise comparison of paired time-dependent AUCs could not be reliably performed due to limited sample size and censoring; the numerical difference is reported descriptively.

Time-dependent ROC analysis was performed to compare the apparent prognostic discrimination of baseline and two-cycle CRAFITY scores. For descriptive comparison of baseline and two-cycle scores, time-dependent AUCs were estimated among patients alive at the 1.5-month landmark, with survival time measured from the landmark. At 6 months, the two-cycle score had an apparent AUC of 0.709 (95% CI 0.586–0.832), and the baseline score had an apparent AUC of 0.645 (95% CI 0.514–0.775). At 12 months, the corresponding apparent AUCs were 0.745 (95% CI 0.658–0.832) and 0.638 (95% CI 0.543–0.733), respectively. The two-cycle score had a numerically higher apparent AUC at both timepoints, but the confidence intervals overlapped. This difference is reported descriptively rather than as formal statistical superiority (Supplementary Table S4). ROC curves are in Figure 4.

To assess incremental prognostic value beyond a clinical reference model (age, sex, BCLC stage, Child–Pugh grade, vascular invasion, treatment line, and concomitant TACE), we compared the time-dependent AUC of the clinical model alone with that of the clinical model plus either the baseline or the two-cycle CRAFITY score (Supplementary Table S5). Adding the two-cycle CRAFITY score increased the 6-month AUC from 0.661 to 0.755 (ΔAUC = 0.094) and the 12-month AUC from 0.699 to 0.811 (ΔAUC = 0.112). Adding the baseline CRAFITY score increased the 6-month AUC from 0.661 to 0.714 (ΔAUC = 0.052) and the 12-month AUC from 0.699 to 0.756 (ΔAUC = 0.057). Formal testing of ΔAUC was not reliably feasible due to the limited sample size and censoring, and these results are therefore reported descriptively.

Calibration of the multivariable model was assessed at 6 and 12 months. The calibration slope was 1.128 at 6 months and 1.186 at 12 months, while the intercept was −0.078 and 0.021, respectively, indicating good agreement between predicted and observed survival (Supplementary Figure S2). The integrated Brier score was 0.150 for the CRAFITY model compared with 0.183 for the reference Kaplan–Meier model, indicating improved prediction accuracy (Supplementary Table S6). These calibration and Brier-score results support internally validated model performance only. They do not establish clinical utility or transportability to other settings.

### 3.7. Sensitivity and Subgroup Analyses

In an exploratory sensitivity analysis excluding all patients who received concomitant TACE during immunotherapy (*n* = 70), the two-cycle CRAFITY score remained independently associated with OS in a multivariable model (HR = 2.68, 95% CI: 1.35–5.32, *p* = 0.005). Detailed multivariable results are presented in Supplementary Table S7. Given the small sample of this subset (*n* = 38, events = 29), this analysis should be considered exploratory and not as definitive evidence against TACE confounding.

In an exploratory subgroup analysis by TKI type, the two-cycle CRAFITY score remained prognostic in both the lenvatinib (*n* = 88; HR 2.10, 95% CI 1.50–2.96, *p* < 0.001) and non-lenvatinib (*n* = 19; HR 3.52, 95% CI 1.32–9.40, *p* = 0.012) subgroups, with a nominally significant interaction (*p* for interaction = 0.041). Given the small non-lenvatinib subgroup and wide confidence interval, this represents an exploratory heterogeneity signal that requires validation in larger cohorts and should not be considered confirmatory. Full results are in Supplementary Table S8.

## 4. Discussion

In this real-world cohort of 108 advanced-HCC patients receiving first-time immune-based combination therapy, the two-cycle CRAFITY score was independently associated with OS (HR 2.36 per 1-point increase, 95% CI 1.70–3.29, *p* < 0.001), with a clear dose–response relationship. Median OS from landmark was 34.6, 12.9, and 6.7 months for scores of 0, 1, and 2, respectively—a difference of nearly 28 months between the lowest- and highest-risk groups. The two-cycle score had apparent AUCs of 0.709 (95% CI 0.586–0.832) at 6 months and 0.745 (95% CI 0.658–0.832) at 12 months, and the baseline score had AUCs of 0.645 (95% CI 0.514–0.775) and 0.638 (95% CI 0.543–0.733), respectively. Adding the two-cycle score to a clinical reference model increased the 12-month AUC from 0.699 to 0.811 (ΔAUC = 0.112), whereas adding the baseline score increased it from 0.699 to 0.756 (ΔAUC = 0.057). Because formal testing of ΔAUC was not reliably feasible, these comparisons are descriptive. The diminishing prognostic gap at 12 months may reflect the influence of subsequent events, including disease progression, post-progression treatments, and cumulative adverse events, which can dilute the prognostic value of early-phase biomarkers. The two-cycle CRAFITY score is therefore most useful for early risk stratification rather than long-term outcome prediction. It should be regarded as a prognostic rather than predictive tool, as defined by prior consensus standards [29], and cannot directly guide treatment modification. The baseline CRAFITY score showed only borderline stratification by log-rank test (OS *p* = 0.07; PFS *p* = 0.09), but was significantly associated with OS in a fully adjusted model (HR 1.54, 95% CI 1.07–2.20, *p* = 0.019). Its discriminative ability was nonetheless lower than that of the two-cycle score (C-index 0.641 vs. 0.706). Calibration of the multivariable model was good, with calibration slopes of 1.128 and 1.186 at 6 and 12 months and an integrated Brier score of 0.150. Bootstrap internal validation suggested good internal consistency (optimism-corrected C-index 0.698). These findings should be interpreted as internally validated model performance rather than proof of clinical utility or transportability. Larger prospective multicenter studies are required to externally validate these findings.

The biological rationale for our findings is straightforward. AFP is a well-established surrogate of tumor burden and proliferation, while CRP is an acute-phase reactant that reflects systemic inflammation driven by the tumor–host immune interaction [30,31]. Two cycles of immunotherapy represent a critical early window during which effective immune activation leads to tumor shrinkage (AFP decline) and resolution of inflammation (CRP decline) [32]. The two-cycle CRAFITY therefore score provides posttreatment prognostic information, integrating both tumor response and inflammatory resolution. In contrast, the baseline score reflects only initial disease severity without accounting for treatment efficacy. This distinction is important because up to one-third of patients exhibit primary resistance to ICIs [7]. Early identification of these patients through the two-cycle CRAFITY score may generate hypotheses for timely treatment modifications.

The failure of the baseline CRAFITY score to significantly stratify OS (*p* = 0.07) warrants discussion. Several factors may explain this discrepancy with the original study by Scheiner et al. [9]. First, population heterogeneity: our patients were predominantly HBV-related HCC from China, whereas the original cohort included a higher proportion of non-viral etiologies from European centers. HCC of different etiologies may exhibit distinct tumor microenvironments and immunobiology, potentially affecting biomarker performance [33]. Second, treatment regimens—our cohort received various PD-1 inhibitors combined with different anti-angiogenic agents (including lenvatinib, sorafenib, apatinib, and bevacizumab), reflecting real-world practice, whereas the original CRAFITY score was primarily developed and validated in patients receiving atezolizumab–bevacizumab [9,18]. The prognostic value of baseline biomarkers may vary across treatment modalities [34]. Heterogeneity of TKIs may have exerted differential effects on AFP suppression and modulation of the inflammatory microenvironment [35,36], potentially influencing the on-treatment changes in AFP and CRP at the two-cycle timepoint. While this heterogeneity limits our ability to attribute biomarker changes solely to immunotherapy, it also enhances the generalizability of our findings to diverse practice settings. Notably, a nominally significant interaction between the two-cycle CRAFITY score and TKI type was observed (*p* for interaction = 0.041), with a larger effect in the non-lenvatinib subgroup (HR 3.52 vs. 2.10 in the lenvatinib subgroup). However, the non-lenvatinib subgroup was small (*n* = 19) with a wide confidence interval, and this interaction should be regarded as an exploratory heterogeneity signal requiring validation in larger cohorts rather than a confirmatory finding. Third, the failure of the baseline CRAFITY score to significantly stratify OS (*p* = 0.07), despite a borderline significant univariable association (HR 1.44, *p* = 0.02), may partially reflect insufficient statistical power related to the single-center sample size (*n* = 108). Larger multicenter studies are needed to definitively assess the baseline score’s independent prognostic value. Importantly, the marked improvement from *p* = 0.07 (baseline) to *p* < 0.0001 (on-treatment) underscores the necessity of on-treatment monitoring rather than abandoning the CRAFITY concept. This observation aligns with the CRAFITY-100 RULE study, in which the baseline CRAFITY score also failed to significantly stratify survival (*p* = 0.267) in a real-world cohort with heterogeneous treatment regimens, further supporting that on-treatment assessment can compensate for the limited discrimination of baseline scores in routine clinical practice. This consistency across both OS and PFS endpoints further reinforces the limited prognostic value of static baseline CRAFITY assessment in real-world heterogeneous cohorts.

Our cohort included patients receiving immune-based combination therapy as either first-line or second-line treatment. Median OS for each CRAFITY risk group stratified by treatment line is summarized in Supplementary Table S2, with corresponding Kaplan–Meier curves in Supplementary Figure S1. The prognostic value of the two-cycle CRAFITY score remained consistent after adjustment for treatment line in the multivariable model (Table 2), suggesting generalizability regardless of treatment line. Treatment line itself was not independently associated with survival (HR 1.40, 95% CI 0.84–2.34, *p* = 0.194), further supporting the utility of the CRAFITY score as a prognostic tool independent of treatment history.

Recent studies have proposed refined models combining baseline CRAFITY with early on-treatment biomarker changes. The CAR grading system (CRAFITY score and AFP response) combined baseline CRAFITY with AFP response, defined as >15% decline within 3 months, showing improved prognostic performance compared with either component alone [18]. The CRAFITY-100 RULE integrated baseline CRAFITY with 4-week AFP/PIVKA-II kinetics and achieved clearer survival stratification than the original score [19]. While these models have demonstrated incremental value, they require more complex calculations, and in some cases additional markers such as PIVKA-II that are not routinely available in all centers. In contrast, our study used the absolute two-cycle CRAFITY score alone, requiring only two routine blood tests (AFP and CRP). This simplicity enhances generalizability and clinical applicability, particularly in resource-limited settings. Our approach also aligns with the standard two-cycle (6–8 week) evaluation timepoint in clinical practice, making it feasible without additional patient visits or costs.

We previously reported that on-treatment PNI provided additional prognostic information beyond baseline PNI in the same cohort [20]. The present study extends this on-treatment assessment paradigm to the CRAFITY score, demonstrating that the additional prognostic information provided by on-treatment assessment is not limited to nutritional-immune markers, but also applies to tumor burden and inflammation markers. These findings suggest that early on-treatment reassessment of multiple biomarker dimensions may provide a comprehensive prognostic profile for patients receiving immunotherapy. Whether combining on-treatment PNI and CRAFITY scores could further improve risk stratification represents an interesting direction for future research.

The simple directional change of the CRAFITY score (improvement vs. non-improvement) was not significantly associated with OS (HR 0.67, 95% CI 0.39–1.15, *p* = 0.142). This may be explained by several factors. First, the binary definition captures any score reduction (e.g., 2 → 1) as “improvement,” yet a patient who improves from 2 to 1 remains in a prognostically unfavorable absolute risk category. The crude categorization fails to distinguish between clinically meaningful reductions (e.g., 2 → 0) and marginal changes. Second, biological homeostasis achieved under treatment may be better reflected by the absolute posttreatment level rather than the magnitude of change, especially for acute-phase reactants like CRP. Third, the limited prognostic power of the baseline CRAFITY score in this cohort inherently constrains any derivative metric. The non-significant result should not be interpreted as evidence that ΔCRAFITY is without value: rather, it suggests that the absolute on-treatment score is a more robust and directly interpretable clinical tool for risk stratification. Future studies with larger samples and more refined definitions of “improvement” (e.g., magnitude of score reduction, or changes in continuous AFP/CRP values) are warranted to further explore the prognostic value of on-treatment CRAFITY changes.

Our findings have potential clinical implications that warrant further investigation. At the two-cycle evaluation timepoint (typically 6–8 weeks after treatment initiation), clinicians routinely order blood tests for safety monitoring. Adding CRAFITY score calculation to this routine blood draw incurs no additional cost or patient burden and provides prognostic information that may support early risk stratification. Patients with a two-cycle CRAFITY score of 2 (median OS 6.7 months) had poorer outcomes than those with a score of 0 (median OS 34.6 months), and may warrant closer clinical monitoring, but no treatment modification can be recommended based solely on this retrospective score. The two-cycle CRAFITY score is intended to serve as an adjunct to radiological assessment, not a replacement. Given the retrospective nature of this study, any treatment modification based on the CRAFITY score—such as treatment intensification or early switching—is hypothesis-generating and requires validation in prospective interventional studies before clinical application.

This study has several limitations. First, this was a single-center, retrospective study with a moderate sample size (*n* = 108, 83 events), which inevitably introduced selection bias. Only patients who completed at least two treatment cycles and had paired AFP and CRP data were included, restricting the analysis to those with relatively good treatment tolerance and early survival. Our findings therefore cannot be generalized to patients who experienced early death or rapid discontinuation before the second cycle. The event-per-variable ratio was approximately 12, which—while adequate for the primary model—limits the robustness of subgroup analyses. Second, our cohort mainly consisted of male Chinese patients with HBV-related HCC, consistent with local epidemiology, but limiting generalizability to non-HBV etiologies, female patients, and other ethnic populations. Third, the study population included both first-line and second-line immunotherapy as well as concomitant TACE, introducing clinical heterogeneity that may influence inflammatory markers and long-term prognosis. Although we performed multivariable adjustment and a TACE-excluded sensitivity analysis, residual confounding cannot be completely excluded. The TACE-excluded sensitivity analysis was limited by the small sample (*n* = 38), and should therefore be considered exploratory. Extrahepatic metastasis and detailed tumor burden were not separately included because BCLC stage already incorporates these dimensions. Adding them would risk model overfitting given the limited events per variable. Prior TACE history was not systematically recorded, and PD-1 inhibitor type was not included as a covariate due to the large number of agents and small sample per agent. These findings should be interpreted in the context of a cohort predominantly treated with PD-1 inhibitor-plus-anti-angiogenic combinations. Validation in other immune-based combination regimens is needed. Fourth, CRP levels may be influenced by intercurrent infections, antibiotic exposure, irAEs, and corticosteroid use. In our clinical practice, patients with uncontrolled active infection at treatment initiation were excluded, and immunotherapy was paused if an intercurrent infection developed. However, antibiotic use, irAE details, and corticosteroid administration during the 6- to 8-week window were not systematically recorded and could not be reliably quantified. Only routine CRP measurements were available, without CRP metabolites or detailed inflammatory profiling. Blood sampling time varied within ±7 days of each cycle, and subsequent treatments after initial immunotherapy discontinuation were not systematically collected. These factors may have influenced biomarker measurements and long-term outcome interpretation. Finally, although the two-cycle CRAFITY score showed apparently better prognostic discrimination at 6 months, this apparent advantage diminished at 12 months. This study provides prognostic risk-stratification evidence only. No treatment modification strategy can be recommended based solely on these retrospective results. Bootstrap internal validation showed good internal consistency, but all estimates were derived from a single-center cohort and require external validation in independent multicenter cohorts before clinical application.

## 5. Future Perspectives

This study demonstrates that the on-treatment CRAFITY score may serve as an early prognostic biomarker for advanced-HCC patients receiving immunotherapy-based combination regimens. However, several unresolved issues remain. For patients with radiologically stable disease at the two-cycle assessment, the current standard of care should be continued regardless of the CRAFITY score. The score should be used only as a prognostic stratification tool to identify high-risk patients who may warrant closer monitoring. It should not justify early treatment modification outside of a clinical trial. Prospective multicenter cohorts are first needed to externally validate the prognostic value and clinical applicability of the two-cycle CRAFITY score. Randomized controlled trials are also warranted in patients with stable disease, but high on-treatment scores, comparing standard continuation versus treatment intensification, to determine whether CRAFITY-guided management improves long-term outcomes. Such studies should incorporate serial radiological and biomarker assessment, as well as standardized documentation of intercurrent infections and immune-related adverse events, to minimize confounding and better distinguish true treatment failure from transient inflammatory fluctuations. Finally, mechanistic studies focusing on inflammatory metabolites, including CRP metabolites, may help reveal the biological mechanisms underlying on-treatment changes in CRP and AFP during immunotherapy.

## 6. Conclusions

This single-center retrospective study suggests that the two-cycle CRAFITY score may provide complementary posttreatment prognostic information in HCC patients who remain evaluable at the two-cycle assessment. The score does not predict treatment benefit, has no established clinical utility for treatment selection, and does not support treatment intensification or early treatment switching. It should be considered an adjunct to imaging, not a replacement. Bootstrap internal validation suggested good internal consistency (optimism-corrected C-index 0.698). Calibration and Brier-score estimates were apparent rather than optimism-corrected, and these findings therefore support internally validated model performance only. They do not establish clinical utility or transportability to other settings. Larger prospective multicenter studies are needed to validate these findings.

## Figures and Tables

**Table 1:** Baseline patient characteristics stratified by two-cycle CRAFITY score.

Characteristic	Overall (*N* = 108)	Two-Cycle CRAFITY 0 (*N* = 37)	Two-Cycle CRAFITY 1 (*N* = 48)	Two-Cycle CRAFITY 2 (*N* = 23)	*p* Value
Age, years (mean ± SD)	56.0 ± 11.6	58.8 ± 8.7	56.3 ± 11.6	51.7 ± 13.7	0.059
Male, *n* (%)	102 (94.4)	35 (94.6)	45 (93.8)	22 (95.7)	0.947
HBV infection, *n* (%)	100 (92.6)	32 (86.5)	46 (95.8)	22 (95.7)	0.217
Cirrhosis, *n* (%)	76 (70.4)	21 (56.8)	37 (77.1)	18 (78.3)	0.082
ECOG PS 1, *n* (%)	20 (18.5)	5 (13.5)	10 (20.8)	5 (21.7)	0.624
BCLC stage C, *n* (%)	96 (88.9)	32 (86.5)	41 (85.4)	23 (100.0)	0.159
Child–Pugh class B, *n* (%)	9 (8.3)	1 (2.7)	6 (12.5)	2 (8.7)	0.268
Vascular invasion, *n* (%)	63 (58.3)	20 (54.1)	24 (50.0)	19 (82.6)	0.027
Extrahepatic metastasis, *n* (%)	41 (38.0)	11 (29.7)	22 (45.8)	8 (34.8)	0.297
Tumor size ≥ 5 cm, *n* (%)	67 (62.0)	18 (48.6)	30 (62.5)	19 (82.6)	0.031
Multiple tumors, *n* (%)	84 (77.8)	27 (73.0)	35 (72.9)	22 (95.7)	0.067
Treatment line, *n* (%)					0.361
First-line	37 (34.3)	14 (37.8)	18 (37.5)	5 (21.7)	
Second-line	71 (65.7)	23 (62.2)	30 (62.5)	18 (78.3)	
Concomitant TACE during immunotherapy, *n* (%)	70 (64.8)	26 (70.3)	33 (68.8)	11 (47.8)	0.156
Baseline AFP, ng/mL (mean ± SD)	5309 ± 12,468	619 ± 2120	4409 ± 10,341	13,382 ± 18,009	<0.001
Baseline CRP, mg/L (mean ± SD)	17.7 ± 25.8	14.5 ± 24.8	15.3 ± 20.5	29.2 ± 35.8	0.068

Values are presented as means ± SD for continuous variables and *n* (%) for categorical variables. *p* values were calculated using ANOVA or Kruskal-Wallis test for continuous variables and chi-squared or Fisher’s exact test for categorical variables, as appropriate. Abbreviations: SD, standard deviation; HBV, hepatitis B virus; PS, performance status; BCLC, Barcelona Clinic Liver Cancer; TACE, transarterial chemoembolization; AFP, alpha-fetoprotein; CRP, C-reactive protein.

**Table 2:** Multivariable Cox regression analysis for overall survival.

Variable	HR (95% CI)	*p* Value
two-cycle CRAFITY score (per 1-point increase)	2.36 (1.70–3.29)	<0.001
Age (per 1-year increase)	1.01 (0.99–1.03)	0.335
Gender (male vs. female)	0.43 (0.17–1.10)	0.077
BCLC stage (C vs. B)	0.56 (0.24–1.30)	0.176
Child–Pugh grade (B vs. A)	1.61 (0.68–3.79)	0.279
Vascular invasion (yes vs. no)	1.17 (0.70–1.96)	0.544
Treatment line (second-line vs. first-line)	1.34 (0.80–2.26)	0.266
TACE (yes vs. no)	0.73 (0.45–1.20)	0.216

Model adjusted for age, gender, BCLC stage, Child–Pugh grade, vascular invasion, treatment line, and TACE. *n* = 108, events = 83. Abbreviations: HR, hazard ratio; CI, confidence interval; BCLC, Barcelona Clinic Liver Cancer.

## Data Availability

Due to the retrospective study design and protection of patient privacy, the raw clinical data supporting the findings of this study are not publicly available. Data can be provided by the corresponding author upon reasonable request and with approval from the Institutional Ethics Committee.

## Supplementary Materials

The following supporting information can be downloaded at https://www.mdpi.com/article/10.3390/curroncol33100598/s1. Table S1: Treatment regimen distribution; Table S2: Median overall survival stratified by two-cycle CRAFITY score and treatment line; Table S3: Objective response rate (ORR) and disease control rate (DCR) by two-cycle CRAFITY score; Table S4: Time-dependent AUC comparison between baseline and two-cycle CRAFITY scores; Table S5: Incremental prognostic value of baseline and two-cycle CRAFITY scores over the clinical reference model; Table S6: Calibration and prediction error of the multivariable model; Table S7: Exploratory multivariable Cox regression analysis, excluding patients who received concomitant TACE during immunotherapy; Table S8: Subgroup Analysis: Prognostic Value of two-Cycle CRAFITY Score Stratified by TKI Type; Figure S1: Kaplan–Meier curves for overall survival stratified by the two-cycle CRAFITY score in subgroups defined by treatment line; Figure S2: Calibration plots at 6 and 12 months.

## Abbreviations

The following abbreviations are used in this manuscript.

HCC	hepatocellular carcinoma
CRP	C-reactive protein
AFP	alpha-fetoprotein
ICI	immune checkpoint inhibitor
OS	overall survival
PFS	progression-free survival
TKI	tyrosine kinase inhibitor
TACE	transcatheter arterial chemoembolization
mRECIST	Modified Response Evaluation Criteria in Solid Tumors
ROC	receiver operating characteristic
AUC	area under the curve
HR	hazard ratio
CI	confidence interval
BCLC	Barcelona Clinic Liver Cancer
NCCN	National Comprehensive Cancer Network
CSCO	Chinese Society of Clinical Oncology
PNI	prognostic nutritional index
