# Supplementary material for: Prognostic Association of the Two-Cycle On-Treatment CRAFITY Score in Advanced HCC: A Single-Center Retrospective Cohort Study
Source: Curr Oncol. 2026 Oct 2;33(10):598. doi: 10.3390/curroncol33100598 (PMC13703901; doi:10.3390/curroncol33100598)
Supplement: Supplementary file 1 [file curroncol-33-00598-s001.zip › curroncol-4521580-supplementary.pdf]

Additional file 1: Supplementary Figures and Tables

Prognostic Association of the Two-Cycle On-Treatment CRAFITY Score in Advanced HCC: A Single-Center Retrospective Cohort Study

Contents

Supplementary Figures (S1, S2)

Supplementary Tables (S1-S8)

Figure S1. Kaplan–Meier curves for overall survival stratified by the two-cycle CRAFITY score in subgroups defined by treatment line

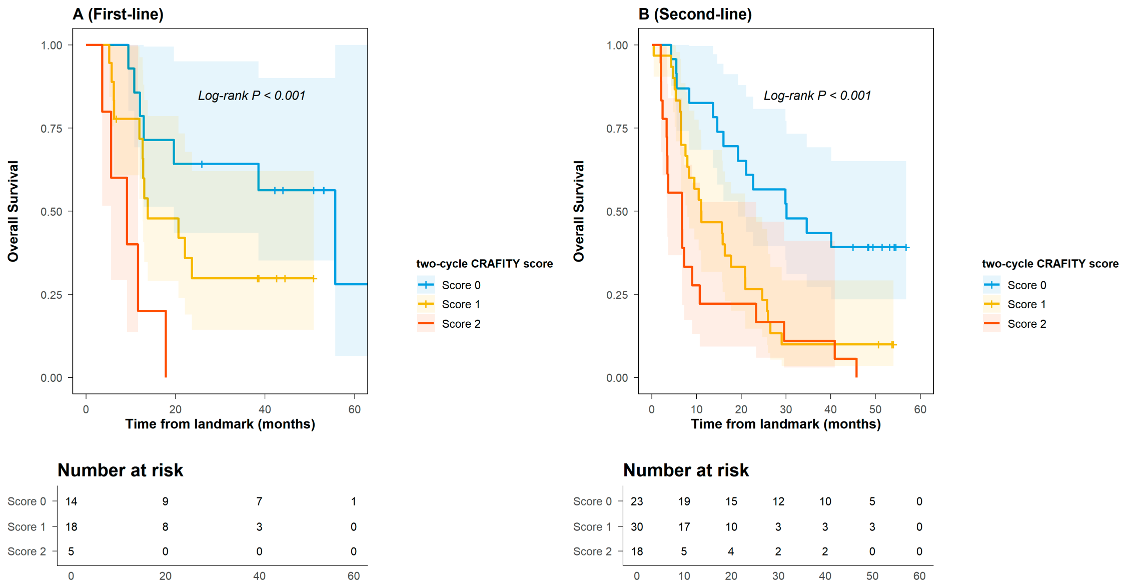

Supplementary Figure S1. Kaplan–Meier curves for overall survival stratified by the two-cycle CRAFITY score in (A) first-line (n = 37) and (B) second-line (n = 71) subgroups, using the landmark time (1.5 months after treatment initiation) as time zero. Median OS from landmark for scores 0, 1, and 2 were 40.4, 13.4, and 9.1 months in the first-line subgroup, and 30.1, 11.0, and 6.7 months in the second-line subgroup. Risk tables below each panel show the number of patients at risk at each timepoint. Shaded areas represent 95% confidence intervals.

Figure S2. Calibration plots at 6 and 12 months.

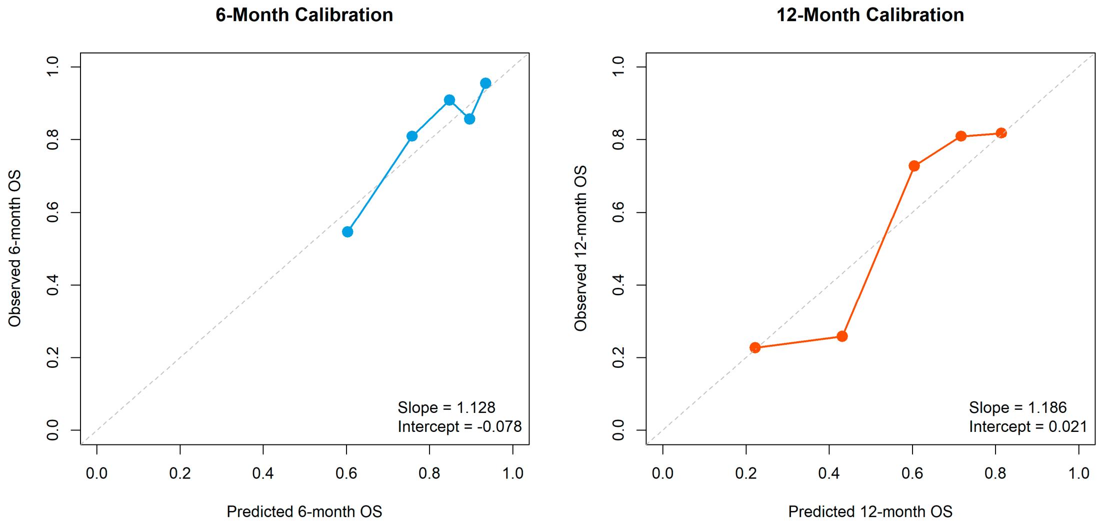

Supplementary Figure S2. Calibration plots of the multivariable model at (A) 6 months and (B) 12 months. The dashed diagonal line represents perfect calibration. The blue and red lines represent the observed calibration of the model. Calibration slope and intercept were 1.128 and −0.078 at 6 months, and 1.186 and 0.021 at 12 months, respectively, indicating good agreement between predicted and observed survival. The Integrated Brier Score was 0.150 for the CRAFITY model, compared with 0.183 for the reference Kaplan–Meier model.

Table S1. Treatment regimen distribution

Regimen	*n* (%)
**PD-1 inhibitor + TKI**	**107 (99.1)**
Camrelizumab + lenvatinib	32 (29.6)
Sintilimab + lenvatinib	28 (25.9)
Tislelizumab + lenvatinib	18 (16.7)
Other PD-1 + TKI combinations	10 (9.3)
Toripalimab + lenvatinib	4 (3.7)
Camrelizumab + sorafenib	4 (3.7)
Sintilimab + sorafenib	4 (3.7)
Sintilimab + apatinib	3 (2.8)
Camrelizumab + apatinib	2 (1.9)
Tislelizumab + sorafenib	2 (1.9)
**PD-L1 inhibitor + anti-VEGF**	**1 (0.9)**
Atezolizumab + bevacizumab	1 (0.9)

Abbreviations: TKI, tyrosine kinase inhibitor; VEGF, vascular endothelial growth factor.

Table S2. Median overall survival stratified by two-cycle CRAFITY score and treatment line.

**Subgroup**	***n***	**CRAFITY 0**	**CRAFITY 1**	**CRAFITY 2**	**Log-rank *P***
First-line combination	37	40.4 months	13.4 months	9.1 months	**< 0.001**
Second-line after TKI failure	71	30.1 months	11.0 months	6.7 months	**< 0.001**
**Overall cohort**	**108**	**34.6 months**	**12.9 months**	**6.7 months**	< 0.001

Median OS was calculated from the landmark time (1.5 months after treatment initiation, approximately corresponding to the completion of 2 treatment cycles). All 108 patients were alive at the landmark and were included in the analysis. P values were calculated using the log-rank test. Due to the limited sample size in each subgroup, these results should be interpreted as exploratory.
Abbreviations: TKI, tyrosine kinase inhibitor; OS, overall survival.

Table S3. Objective response rate (ORR) and disease control rate (DCR) by two-cycle CRAFITY score

**CRAFITY Score**	***N***	**ORR**	**DCR**
0	37	5/37 (13.5%)	35/37 (94.6%)
1	48	5/48 (10.4%)	37/48 (77.1%)
2	23	2/23 (8.7%)	16/23 (69.6%)
P for trend	—	0.549	**0.011**

Response evaluated by mRECIST. P for trend calculated using the Cochran-Armitage trend test.

Bold value indicates statistical significance ($P < 0.05$).

Abbreviations: ORR, objective response rate; DCR, disease control rate.

Table S4. Time-Dependent AUC Comparison Between Baseline and two-Cycle CRAFITY scores.

Timepoint	Baseline CRAFITYAUC (95% CI)	2-Cycle CRAFITYAUC (95% CI)
6 months	0.645 (0.514–0.775)	0.709 (0.586–0.832)
12 months	0.638 (0.543–0.733)	0.745 (0.658–0.832)

AUC values are reported with 95% confidence intervals for each score separately. Formal pairwise comparison of paired time-dependent AUCs could not be reliably performed due to the limited sample size and censoring; the numerical difference is reported descriptively.

Table S5. Incremental prognostic value of baseline and two-cycle CRAFITY scores over the clinical reference model.

Score	Timepoint	Clinical Model AUC (95% CI)	Extended model AUC (95% CI)	ΔAUC
Baseline CRAFITY	6 months	0.661 (0.527–0.796)	0.714 (0.596–0.831)	0.052
Baseline CRAFITY	12 months	0.699 (0.594–0.803)	0.756 (0.663–0.848)	0.057
two-cycle CRAFITY	6 months	0.661 (0.527–0.796)	0.755 (0.628–0.882)	0.094
two-cycle CRAFITY	12 months	0.699 (0.594–0.803)	0.811 (0.724–0.898)	0.112

Clinical reference model: age, sex, BCLC stage, Child–Pugh grade, vascular invasion, treatment line, and concomitant TACE. Extended models additionally include either the baseline or two-cycle CRAFITY score. ΔAUC was calculated as the difference between extended and clinical model AUC values. Formal testing of ΔAUC was not reliably feasible due to limited sample size and censoring; results are reported descriptively. AUC, area under the curve; CI, confidence interval.

Table S6. Calibration and Prediction Error of the Multivariable Model

Timepoint	Calibration slope	Calibration intercept	Integrated Brier score (IBS)
6 months	1.128	−0.078	—
12 months	1.186	0.021	—
Overall (0–63.9 months)	—	—	0.150

Reference Kaplan–Meier model IBS: 0.183. Calibration slope and intercept were estimated using logistic calibration regression on quintile groups. Lower Brier score indicates better prediction

accuracy.

Table S7. Exploratory multivariable Cox regression analysis excluding patients who received concomitant TACE during immunotherapy.

Variable	HR (95% CI)	P value
two-cycle CRAFITY score (per 1-point increase)	2.68 (1.35–5.32)	0.005
Age (per 1-year increase)	1.00 (0.96–1.04)	0.984
Gender (male vs female)	1.24 (0.14–11.12)	0.847
BCLC stage (C vs B)	0.24 (0.03–2.10)	0.197
Child-Pugh grade (B vs A)	3.77 (1.05–13.49)	0.041
Vascular invasion (yes vs no)	1.10 (0.41–2.94)	0.848
Treatment line (second-line vs first-line)	13.67 (1.58–118.22)	0.018

Model adjusted for age, gender, BCLC stage, Child-Pugh grade, vascular invasion, and treatment line. This analysis excluded 70 patients who received concomitant TACE during immunotherapy, leaving 38 patients (29 events). Due to the small sample size, this analysis is exploratory and should not be considered confirmatory.

Abbreviations: TACE, transarterial chemoembolization; HR, hazard ratio; CI, confidence interval; BCLC, Barcelona Clinic Liver Cancer.

Table S8. Subgroup Analysis: Prognostic Value of two-Cycle CRAFITY Score Stratified by TKI Type

Subgroup	n	Events	HR (95% CI) per 1-point increase	P value
Lenvatinib	88	69	2.10 (1.50–2.96)	< 0.001
Other TKI (sorafenib/apatinib/regorafenib)	19	14	3.52 (1.32–9.40)	0.012
P for interaction				0.041

Model: univariable Cox regression within each subgroup; interaction tested in the pooled TKI cohort (*n* = 107, excluding one patient receiving atezolizumab plus bevacizumab).

Abbreviations: TKI, tyrosine kinase inhibitor; HR, hazard ratio; CI, confidence interval.
